# Safety evaluation of cinacalcet: Signal mining and analysis of adverse events based on the FAERS database

**DOI:** 10.1371/journal.pone.0331510

**Published:** 2025-10-27

**Authors:** Junxia Cao, Na Yang, Cheng Huang

**Affiliations:** 1 Guizhou Nursing Vocational College, Guiyang, China; 2 Department of Comprehensive Ward, The Affiliated Hospital of Guizhou Medical University, Guiyang, China; Azienda Ospedaliera Universitaria Integrata Verona, ITALY

## Abstract

**Objective:**

This study utilized the FDA Adverse Event Reporting System (FAERS) to assess signals of adverse events (AEs) associated with cinacalcet, aiming to enhance its safe and rational clinical application.

**Methods:**

Adverse event reports related to cinacalcet were extracted from the FAERS database from the first quarter of 2004 to the first quarter of 2025. The AE reports were categorized by Preferred Terms (PTs) and System Organ Classes (SOCs), and risk signals were analyzed using disproportionality analysis.

**Results:**

Among 30,540 AE reports where cinacalcet was the primary suspect drug, females exhibited a higher reporting frequency than males (47.30% vs. 39.80%). The highest proportion of reports was observed in the 60–74 age group (23.85%). Most AEs predominantly occurred within <7 days (11.14%) or ≥60 days (26.35%) of drug administration. A total of 78 significant PT signals were detected, including known AEs such as nausea, vomiting, loss of appetite, abdominal discomfort, hypocalcemia and epigastric pain, aligning with the drug’s prescribing information. Additionally, several AEs previously undocumented in the drug’s specifications were observed, including precocious puberty, parathyroid hemorrhage, hypoproteinemia, pancreatic atrophy, monocytopenia, cardiac death and arrhythmia.

**Conclusion:**

Patient evaluation should be conducted prior to the clinical use of cinacalcet, particularly for individuals with heart failure, hepatic or renal insufficiency, and hypocalcemia. Close monitoring of electrolytes and vigilance for gastrointestinal, cardiovascular, and endocrine-related AEs are recommended. Prompt interventions should be implemented in cases of adverse reactions or disease progression to prevent serious complications or deterioration.

## 1. Introduction

The parathyroid gland is a vital endocrine organ in the human body. Parathyroid hormone (PTH), which is secreted by the parathyroid gland, plays a crucial role in regulating bone, nervous, digestive, and other physiological systems. Consequently, parathyroid gland disorders are often described as having a characteristic of “small lesions with big consequences” particularly in the context of hyperparathyroidism (HPT) [1].

Hyperparathyroidism (HPT) is a group of endocrine disorders caused by increased active or passive secretion of parathyroid hormone (PTH) from the parathyroid glands. The condition is primarily characterized by disturbances in calcium, phosphorus, and bone metabolism. HPT can be classified into three categories based on its etiology: primary hyperparathyroidism (PHPT), secondary hyperparathyroidism (SHPT), and tertiary hyperparathyroidism (THPT) [2]. PHPT is commonly caused by parathyroid hyperplasia, adenomas, or adenocarcinomas, whereas SHPT typically results from chronic hypocalcemia due to prolonged vitamin D deficiency, malabsorption syndromes or renal insufficiency. Economic development and lifestyle changes have contributed to a rising incidence of hyperparathyroidism [3].

Cinacalcet is an approved medication for treating hyperparathyroidism. As an allosteric calcium-sensing receptor (CaSR) modulator, it enhances the sensitivity of CaSR to serum calcium, thereby reducing parathyroid hormone (PTH) secretion, promoting renal calcium excretion, and ultimately lowering serum calcium levels [4]. The drug has been approved by the European Medicines Agency (EMA) and the U.S. Food and Drug Administration (FDA) for the treatment of hypercalcemia in adults who are not candidates for parathyroidectomy [3]. Additionally, cinacalcet has been approved by the EMA for the treatment of SHPT in children aged ≥3 years on dialysis [5]. However, initial studies on its pediatric use have primarily been limited to case reports or small, single-center observational studies [5–7]. These studies often have strict inclusion criteria, are conducted under controlled conditions, and feature relatively short trial durations, making it challenging to identify rare or long-term adverse events associated with cinacalcet use. Consequently, these studies may not fullyreflect the drug’s real-world performance in broader populations. To address these gaps, it is essential to gather safety data for cinacalcet from real-world evidence.

The U.S. FDA Adverse Event Reporting System (FAERS) is a pharmacovigilance database that systematically collects spontaneously reported adverse drug events (AEs) from healthcare institutions, physicians, healthcare professionals, patients, and other stakeholders, and it serves as a crucial source of evidence for detecting adverse drug events during drug safety evaluation [8] Due to its large volume of data, diverse information, standardization, and public accessibility, FAERS has become an indispensable tool for pharmacovigilance research and drug safety surveillance [9]. By conducting an in-depth analysis of the mined data, researchers can obtain a more comprehensive understanding of the safety profiles and adverse reaction characteristics of drugs in clinical practice. In this study, we conducted a comprehensive evaluation of the safety of cinacalcet by analyzing AE reports from the FAERS database. The primary objective was to identify potential safety concerns associated with cinacalcet, thereby providing valuable insights and references to guide clinical practice and enhance medication safety.

## 2. Data sources

The adverse event (AE) data analyzed in this study were obtained from the U.S. Food and Drug Administration Adverse Event Reporting System (FAERS) database, a publicly available pharmacovigilance database. It aims to collect information on adverse events following drug approval, including seven sections: patient outcomes information (OUTC), demographic and administrative information (DEMO), drug information (DRUG), indications for drug administration (INDI), adverse drug reaction information (REAC), reported sources (RPSR), and drug therapy start and end dates (THER) [10]. As all records in this database have been completely de-identified, researchers could not access any information that could potentially identify individuals (including both direct and indirect identifiers) during data collection and analysis. Since the data were collected retrospectively for regulatory purposes and did not involve direct participation of human subjects, this study did not require approval from an ethics committee. Considering the marketing timeline of cinacalcet, all adverse events (AEs) data related to cinacalcet from the first quarter of 2004 to the first quarter of 2025 were extracted from the FAERS database.

## 3. Data cleaning and analysis

### 3.1. Standardization of drug names and adverse drug reactions

**Fig 1:**
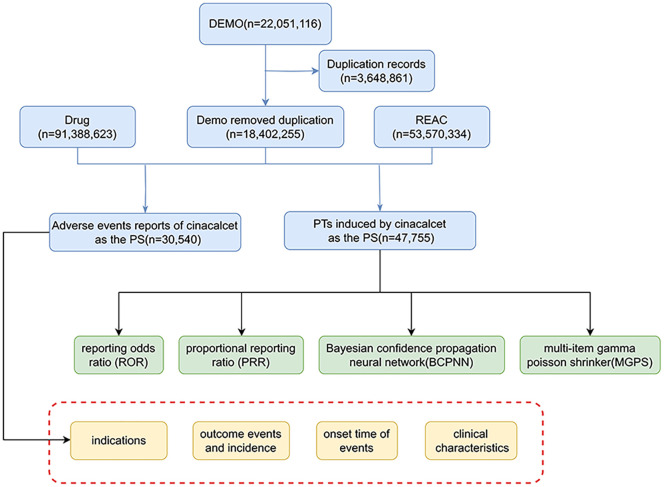
The flow diagram of selecting cinacalcet-related AEs from FAERS database. This figure illustrates the data extraction and filtering process for identifying cinacalcet-associated adverse events from the FDA Adverse Event Reporting System (2004Q1-2025Q1).

The search was performed using the generic drug names “Cinacalcet (brand name Sensipar)” as the target terms (S1 Table). For records with identical case numbers in the DEMO table, only the most recent report (based on the reporting date) was retained, while redundant entries were removed to ensure clean and standardized data collection. Reports involving cinacalcet were categorized into four groups based on its role in the reported AEs: primary suspect (PS), secondary suspect (SS), concomitant (C), and interacting (I). This study focused primarily on reports where cinacalcet was designated as the PS drug. Adverse events were coded using Preferred Terms (PTs) from the latest version of the Medical Dictionary for Regulatory Activities(MedDRA v25.0). The corresponding System Organ Classes (SOCs) were also documented to facilitate statistical analysis and categorization of the data. Additionally, the clinical characteristics of patients experiencing cinacalcet-associated AEs were collected, including sex, age, reporter type, region, treatment outcomes, and time to onset (TTO). A comprehensive flow diagram illustrating the data cleaning and analysis process is presented in Fig 1.

### 3.2. Signal analysis algorithms and statistical methods

This study utilized the four-fold table proportional imbalance method, a technique widely adopted in pharmacovigilance research, to conduct disproportionality analysis examining potential associations between cinacalcet and all reported adverse events (AEs). Four primary metrics were calculated using standard formulae: the Reporting Odds Ratio (ROR), the Proportional Reporting Ratio (PRR), the Information Component (IC) from the Bayesian Confidence Propagation Neural Network (BCPNN), and the Empirical Bayesian Geometric Mean (EBGM) from the Multi-item Gamma Poisson Shrinker (MGPS). These measures were employed to comprehensively evaluate potential correlations between cinacalcet and the reported adverse events [11,12]. The principles of the four methods are as follows: the ROR is calculated as the odds of reporting a specific adverse event (AE) with the target drug compared to all other drugs in the FAERS database; the PRR compares the proportion of reports for a specific AE with the target drug to the proportion of the same AE with all other drugs; the IC employs a Bayesian approach to compare the observed and expected numbers of AE reports; and the EBGM, as an advanced Bayesian method, calculates the observed-to-expected AE reporting ratios while adjusting for reporting variability [11,12]. Given ROR’s methodological transparency, interpretability of results, and widespread application in pharmacovigilance research, we designated it as the primary analytical metric in this study [13,14]. Statistical significance was assessed using the χ² test on a fourfold table, following which the obtained P-values were adjusted for multiple comparisons with the false discovery rate (FDR) method. An adjusted P-value (p-adj) < 0.05 was defined as the threshold for statistical significance [13,14]. Results from other algorithms were used as supplementary analyses to evaluate the consistency and stability of the primary signals. This approach aimed to highlight key analytical focus, enhance the readability of the results, and ensure the reliability of the conclusions [13,14]. By adjusting thresholds and variances, the methods were also optimized to capture rare potential adverse events.The principle underlying all these algorithms is based on the classical fourfold table, which analyzes the association between a drug and an adverse event (AE) by comparing observed frequency ratios in exposed and unexposed populations (Table 1). The formulas and thresholds for each algorithm are detailed in Table 2.

**Table 1:** Fourfold table of disproportionality method.

Types of Medication	Number of AE Reports for Cinacalcet	Number of AE Reports for Non-Cinacalcet	Total
Cinacalcet	a	b	a + b
Non-cinacalcet	c	d	c + d
Total	a + c	b + d	N = a + b + c + d

**Table 2:** ROR, PRR, BCPNN, and EBGM methods, formulas, and thresholds.

Method	Formula	Threshold
ROR	$\text{ROR}=\frac{\text{a}/\text{c}}{\text{b}/\text{d}}$	a ≥ 3 95%CI (lower limit) > 1
	$\text{SE}(\text{lnROR})=\sqrt{\frac{\text{1}}{\text{a}}+\frac{\text{1}}{\text{b}}+\frac{\text{1}}{\text{c}}+\frac{\text{1}}{\text{d}}}$	
	$\text{95}\%\text{CI}={\text{e}}^{\text{ln}(\text{ROR})\pm \text{1}.\text{96se}}$	
PRR	$\text{PRR}=\frac{\text{a}/(\text{a}+\text{b})}{\text{c}/(\text{c}+\text{d})}$	a ≥ 3 95%CI (lower limit) > 1
	$\text{SE}(\text{lnPRR})=\sqrt{\frac{\text{1}}{\text{a}}-\frac{\text{1}}{\text{a}+\text{b}}+\frac{\text{1}}{\text{c}}-\frac{\text{1}}{\text{c}+\text{d}}}$	
	$\text{95}\%\text{CI}={\text{e}}^{\text{ln}(\text{PRR})\pm \text{1}.\text{96se}}$	
BCPNN	$\text{IC}={\text{log}}_{\text{2}}\frac{\text{p}(\text{x},\text{y})}{\text{p}(\text{x}textrmp(\text{y})}={\text{log}}_{\text{2}}\frac{\text{a}(\text{a}+\text{b}+\text{c}+\text{d})}{\left(\text{a}+\text{b})(\text{a}+\text{c}\right)}$	IC025 > 0
	$\text{E}(\text{IC})={\text{log}}_{\text{2}}\frac{\left(\text{a}+\gamma \text{11})(\text{a}+\text{b}+\text{c}+\text{d}+\alpha )(\text{a}+\text{b}+\text{c}+\text{d}+\beta \right)}{\left(\text{a}+\text{b}+\text{c}+\text{d}+\gamma )(\text{a}+\text{b}+\alpha \text{1})(\text{a}+\text{c}+\beta \text{1}\right)}$	
	$\text{V}(\text{IC})=\frac{\text{1}}{{\left(\text{ln2}\right)}^{\text{2}}}[\frac{(\text{a}+\text{b}+\text{c}+\text{d})-\text{a}+\gamma -\gamma \text{11}}{\left(\text{a}+\gamma \text{11})(\text{1}+\text{a}+\text{b}+\text{c}+\text{d}+\gamma \right)}+\frac{(\text{a}+\text{b}+\text{c}+\text{d})-(\text{a}+\text{b})+\text{a}-\alpha \text{1}}{\left(\text{a}+\text{b}+\alpha \text{1})(\text{1}+\text{a}+\text{b}+\text{c}+\text{d}+\alpha \right)}+\frac{(\text{a}+\text{b}+\text{c}+\text{d}+\alpha )-(\text{a}+\text{c})+\beta -\beta \text{1}}{\left(\text{a}+\text{b}+\beta \text{1})(\text{1}+\text{a}+\text{b}+\text{c}+\text{d}+\beta \right)}]$	
	$\gamma =\gamma \text{11}\frac{\left(\text{a}+\text{b}+\text{c}+\text{d}+\alpha )(\text{a}+\text{b}+\text{c}+\text{d}+\beta \right)}{\left(\text{a}+\text{b}+\alpha \text{1})(\text{a}+\text{c}+\beta \text{1}\right)}$	
	$\text{IC}-\text{2SD}=\text{E}(\text{IC})-\text{2}\sqrt{\text{V}(\text{IC})}$	
MGPS	$\text{EBGM}=\frac{\text{a}(\text{a}+\text{b}+\text{c}+\text{d})}{\left(\text{a}+\text{c})(\text{a}+\text{b}\right)}$	EBGM05 > 1
	$\text{SE}(\text{lnEBGM})=\sqrt{\frac{\text{1}}{\text{a}}+\frac{\text{1}}{\text{b}}+\frac{\text{1}}{\text{c}}+\frac{\text{1}}{\text{d}}}$	
	$\text{95}\%\text{CI}={\text{e}}^{\text{ln}(\text{EBGM})\pm \text{1}.\text{96se}}$	

A larger 95% confidence interval (CI) value indicates stronger signal intensity, suggesting a higher likelihood of a statistical association between the target drug and the AE [15]. Statistical analyses were performed using R software(version 4.4.0), and used ggplot2 for forest plot visualization.

## 4. Results

### 4.1. Basic information about cinacalcet-related adverse events

From the first quarter of 2004 to the first quarter of 2025, this study collected a total of 22,051,116 adverse drug event reports. After removing duplicate data, 18,402,255 reports were obtained. Among these, 30,540 reports identified cinacalcet as the primary suspected drug. Since a single report may include multiple adverse events, the total number of adverse events related to cinacalcet was 47,755. In all AE reports involving cinacalcet, for gender-specific comparisons, this study exclusively utilized data from individuals with documented gender (male and female). The proportion of female patients was higher than males (47.30% vs 39.80%). Regarding age distribution, 32.91% of the reports lacked age information. Among the reports with clear age data, the median age and interquartile range for AEs of cinacalcet were 60.00 (50.00, 70.00) years.

Further analysis revealed that the incidence of AEs was highest in the 60–74 age group (23.85%), followed by the 45–59 age group (21.40%) and the 18–44 age group (10.76%). These findings indicate that cinacalcet-related AEs predominantly occurred in middle-aged and elderly populations. In addition, in terms of the source of reports, the majority of reports came from other health professionals (32.48%), followed by consumers (30.28%), pharmacists (25.16%) and physicians (11.35%). Geographically, the majority of reports originated from the United States (89.52%). Regarding clinical outcomes, unspecified serious adverse events accounted for the largest proportion (43.19%). Among specified outcomes, AEs leading to hospitalization were the most common (31.57%), followed by death (18.55%), disability (3.53%), and life-threatening events (3.16%). Finally, for time to onset (TTO) of AEs, the median time and interquartile range for the occurrence of adverse events were 82.00 (8.00, 314.75) days. Most AEs occurred within <7 days (11.14%) or ≥60 days (26.35%) after drug administration. Detailed information is provided in Table 3.

**Table 3:** Characteristics of AE reports associated with cinacalcet.

Basic Information	Categories	Reported cases (%)
sex	female	14444(47.30)
	male	12154(39.80)
	unknown	3942(12.91)
Age/yr		60.00(50.00,70.00)^a^
	<18	139(0.46)
	18-44	3286(10.76)
	45-59	6537(21.40)
	60-74	7285(23.85)
	75-89	3022(9.90)
	>=90	219(0.72)
	unknow	10052(32.91)
Reporter	Other health-professional	9919(32.48)
	Consumer	9246(30.28)
	Pharmacist	7683(25.16)
	Physician	3467(11.35)
	unknown	224(0.73)
Reported countries	United States	25786(89.52)
	other	3019(10.48)
Outcomes	other serious	5422(43.19)
	hospitalization	3963(31.57)
	death	2328(18.55)
	disability	443(3.53)
	life threatening	397(3.16)
TTO/Day		82.00(8.00,314.75)^a^
	<7	537(11.14)
	7 ~ 28	295(6.12)
	28 ~ 60	235(4.88)
	>=60	1270(26.35)
	unknow	2483(51.51)

^a^Continuous variables are expressed as medians.

The year with the fewest reports was 2004 (97 reports), while the year with the most reports was 2017 (7,834 reports). From 2015 to 2018, the number of adverse event reports reached a record high (S2 Table).

### 4.2. Signal detection based on system organ classification level (SOC)

**Fig 2:**
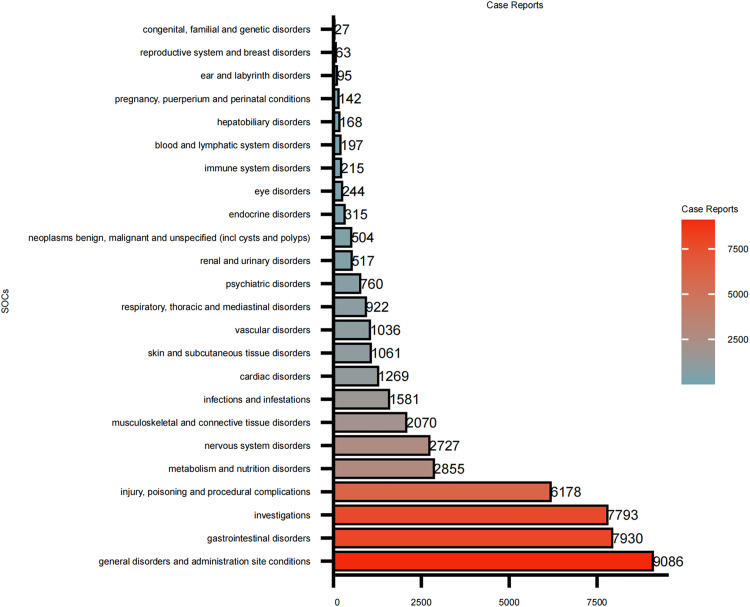
The case reports of AEs of cinacalcet at the SOC level in FAERS. Distribution of reported adverse events across organ systems, showing the number of events for each SOC category.

**Fig 3:**
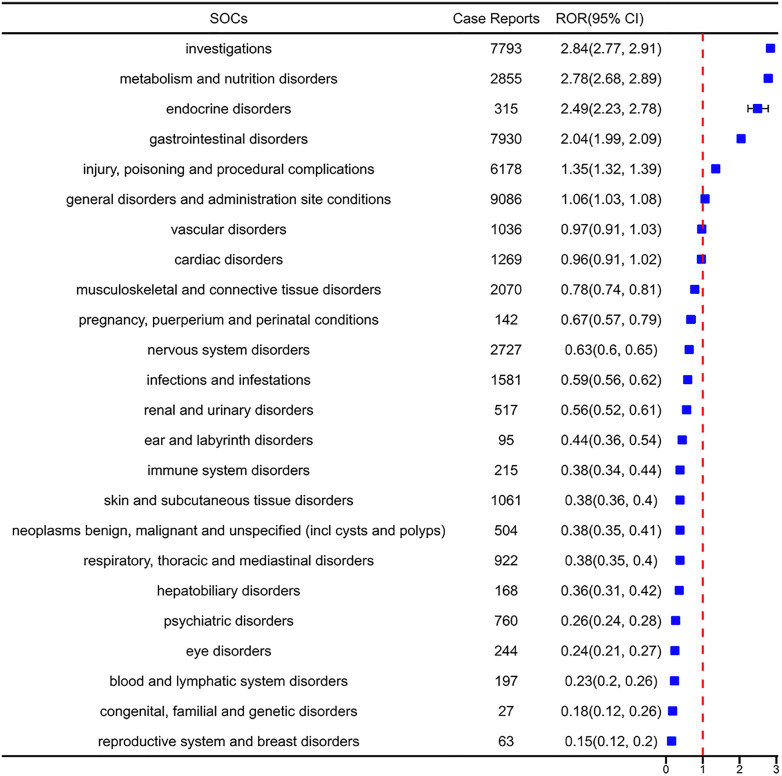
The SOCs according to the top 24 confidence levels in the ROR. Ranking of organ systems based on signal strength measured through disproportionality analysis (ROR method); A signal is considered significant if the number of reports (a) is ≥ 3 and the lower bound of the ROR’s 95% CI is greater than 1.

This study analyzed the adverse events (AEs) of cinacalcet and found that they involved 24 SOCs. By combining four different algorithms for analysis, positive signals were identified for certain SOCs. Ranking was performed based on the number of adverse event reports (Fig 2) and the strength of the ROR signals (Fig 3) respectively, including: Gastrointestinal disorders (n = 7930, ROR2.04, PRR1.86, IC0.9, EBGM1.86), Investigations (n = 7793, ROR2.84, PRR2.54, IC1.34, EBGM2.53), Metabolism and nutrition disorders (n = 2855, ROR2.78, PRR2.68, IC1.42, EBGM2.67), Endocrine disorders (n = 315, ROR2.49, PRR2.48, IC1.31, EBGM2.47), Injury, poisoning, and procedural complications (n = 6178, ROR1.35, PRR1.31, IC0.38, EBGM1.31), and General disorders and administration site conditions (n = 9086, ROR1.06, PRR1.05, IC0.07, EBGM1.05). All detailed information can be found in S3 Table.

The SOCs of gastrointestinal disorders, investigations, and metabolism and nutrition disorders were consistent with common adverse events described in the drug’s prescribing information, indicating that the data obtained in this study are highly reliable.Notably, some SOCs, such as endocrine disorders, included adverse events that are not mentioned in the drug package insert. These findings highlight the need for further investigation and attention to these potential safety signals.

### 4.3. Signal detection based on preferred term (PT)

At the preferred term (PT) level, we applied four algorithms to analyze adverse event signals and assess adherence to various screening criteria, after excluding diseases related to “musculoskeletal and connective tissue disorders, neoplasms benign, malignant and unspecified (incl. cysts and polyps), renal and urinary disorders, vascular disorders, pregnancy, puerperium and perinatal conditions, and nervous system disorders” (as these conditions may be attributed to the progression of the primary disease), resulting in 78 PTs (Table 4).

**Table 4:** Signal strength of cinacalcet-related adverse-event reports at the Preferred Term level in the FAERS database.

SOCs/PTs	Case Reports	ROR(95% CI)	PRR(95% CI)	χ2	IC(IC025)	EBGM(EBGM05)	p.value	p.adj
**Investigations**								
blood parathyroid hormone abnormal	340	1390.56(1186.07, 1630.29)	1380.66(1180.29, 1615.04)	210024.98	9.27(9.08)	619.16(542.01)	<0.001	<0.001
blood parathyroid hormone increased	581	307.59(280.52, 337.27)	303.86(275.49, 335.15)	137981.92	7.9(7.77)	239.26(221.51)	<0.001	<0.001
adjusted calcium decreased	20	299(182.56, 489.69)	298.87(183.1, 487.85)	4687.53	7.88(7.2)	236.16(156.29)	<0.001	<0.001
laboratory test abnormal	4214	226.53(218.92, 234.41)	206.63(198.69, 214.89)	728464.96	7.45(7.4)	174.62(169.7)	<0.001	<0.001
blood parathyroid hormone decreased	202	187.9(161.85, 218.14)	187.1(159.95, 218.86)	32043.72	7.33(7.11)	160.48(141.64)	<0.001	<0.001
blood calcium abnormal	140	115.29(96.86, 137.22)	114.95(96.36, 137.13)	14343.69	6.71(6.46)	104.35(90.2)	<0.001	<0.001
blood phosphorus abnormal	39	114.82(82.57, 159.65)	114.72(82.21, 160.08)	3988.39	6.7(6.23)	104.16(79.06)	<0.001	<0.001
blood 1,25-dihydroxycholecalciferol increased	5	74.73(30.22, 184.78)	74.72(30.33, 184.08)	340.93	6.13(4.94)	70.11(32.87)	<0.001	<0.001
fibroblast growth factor 23 increased	3	74.72(23.22, 240.46)	74.72(23.05, 242.2)	204.56	6.13(4.66)	70.11(26.37)	<0.001	<0.001
blood phosphorus increased	119	57.53(47.84, 69.18)	57.39(48.11, 68.46)	6272.49	5.77(5.51)	54.64(46.83)	<0.001	<0.001
blood calcium increased	289	41.86(37.21, 47.1)	41.62(37, 46.81)	11047.71	5.33(5.16)	40.16(36.39)	<0.001	<0.001
blood calcium decreased	345	38.07(34.19, 42.4)	37.8(34.27, 41.69)	11960.76	5.19(5.04)	36.6(33.45)	<0.001	<0.001
calcium ionised decreased	6	30.29(13.46, 68.17)	30.29(13.56, 67.65)	165.47	4.88(3.8)	29.52(14.98)	<0.001	<0.001
angiotensin converting enzyme increased	3	21.83(6.96, 68.45)	21.83(7, 68.04)	58.5	4.42(2.99)	21.44(8.24)	<0.001	0.01
blood 25-hydroxycholecalciferol decreased	5	20.6(8.51, 49.9)	20.6(8.53, 49.76)	91.57	4.34(3.17)	20.25(9.66)	<0.001	<0.001
blood phosphorus decreased	38	13.05(9.48, 17.97)	13.04(9.53, 17.84)	417.61	3.69(3.23)	12.9(9.87)	<0.001	<0.001
urine calcium increased	3	11.56(3.7, 36.04)	11.55(3.71, 36)	28.63	3.52(2.09)	11.45(4.42)	0.002	0.006
blood viscosity decreased	3	10.88(3.49, 33.93)	10.88(3.49, 33.91)	26.66	3.43(2.01)	10.79(4.17)	0.003	0.007
body temperature abnormal	9	6.27(3.26, 12.07)	6.27(3.28, 11.97)	39.64	2.64(1.74)	6.24(3.61)	<0.001	<0.001
vitamin d increased	3	6.14(1.97, 19.08)	6.14(1.97, 19.14)	12.83	2.61(1.19)	6.11(2.36)	0.014	0.029
human chorionic gonadotropin increased	4	5.78(2.16, 15.43)	5.78(2.17, 15.4)	15.72	2.52(1.26)	5.75(2.53)	0.006	0.014
protein total decreased	18	5.74(3.61, 9.12)	5.74(3.59, 9.19)	70.06	2.51(1.86)	5.71(3.88)	<0.001	<0.001
blood pressure abnormal	59	3.75(2.9, 4.84)	3.74(2.9, 4.83)	118.2	1.9(1.53)	3.73(3.01)	<0.001	<0.001
electrocardiogram qt prolonged	92	3.16(2.57, 3.87)	3.15(2.59, 3.83)	134.9	1.65(1.36)	3.15(2.65)	<0.001	<0.001
blood albumin decreased	20	3.04(1.96, 4.71)	3.04(1.98, 4.68)	27.28	1.6(0.98)	3.03(2.1)	<0.001	<0.001
**Metabolism and Nutrition Disorders**								
calciphylaxis	147	103.94(87.76, 123.1)	103.62(86.86, 123.61)	13675.52	6.57(6.33)	94.93(82.4)	<0.001	<0.001
calcification metastatic	3	42.03(13.27, 133.1)	42.03(13.22, 133.59)	115.82	5.34(3.89)	40.55(15.46)	<0.001	<0.001
hypocalcaemia	610	41.31(38.08, 44.81)	40.79(37.71, 44.12)	22855.29	5.3(5.18)	39.4(36.81)	<0.001	<0.001
hypercalcaemia	289	29.41(26.16, 33.06)	29.24(26, 32.89)	7682.32	4.83(4.67)	28.52(25.86)	<0.001	<0.001
hyperphosphataemia	38	23.43(16.99, 32.31)	23.41(17.11, 32.03)	798.68	4.52(4.06)	22.95(17.54)	<0.001	<0.001
hypophagia	305	15.18(13.55, 17)	15.09(13.42, 16.97)	3960.1	3.9(3.73)	14.9(13.55)	<0.001	<0.001
tetany	18	11.88(7.46, 18.9)	11.87(7.42, 19)	177.37	3.56(2.9)	11.76(7.97)	<0.001	<0.001
appetite disorder	55	10.82(8.3, 14.12)	10.81(8.22, 14.22)	485.05	3.42(3.04)	10.72(8.58)	<0.001	<0.001
iron overload	6	6.66(2.98, 14.86)	6.66(2.98, 14.88)	28.68	2.73(1.66)	6.62(3.38)	<0.001	0.001
hypermagnesaemia	3	5.81(1.87, 18.06)	5.81(1.86, 18.11)	11.88	2.53(1.11)	5.78(2.24)	0.016	0.032
hypochloraemia	5	5.36(2.23, 12.9)	5.36(2.22, 12.95)	17.64	2.42(1.26)	5.34(2.56)	0.003	0.007
hypoproteinaemia	8	5.2(2.6, 10.41)	5.2(2.62, 10.33)	27	2.37(1.43)	5.18(2.9)	<0.001	0.001
decreased appetite	813	4.41(4.12, 4.73)	4.35(4.1, 4.61)	2100.23	2.12(2.02)	4.34(4.1)	<0.001	<0.001
hypophosphataemia	23	3.88(2.58, 5.85)	3.88(2.57, 5.86)	49.05	1.95(1.37)	3.87(2.75)	<0.001	<0.001
**Endocrine Disorders**								
parathyroid haemorrhage	9	2522.22(776.69, 8190.59)	2521.74(777.98, 8173.92)	6977.75	9.6(8.43)	776.61(289.86)	<0.001	<0.001
parathyroid hyperplasia	24	996.74(575.11, 1727.5)	996.24(575.47, 1724.67)	12632.77	9.04(8.36)	527.89(333.2)	<0.001	<0.001
hyperparathyroidism tertiary	21	500.99(299.48, 838.09)	500.77(300.83, 833.59)	7239.56	8.44(7.74)	346.43(225.24)	<0.001	<0.001
parathyroid cyst	4	249.08(84.29, 736.01)	249.06(84.75, 731.94)	808.58	7.67(6.28)	203.96(82.38)	<0.001	<0.001
parathyroid gland enlargement	16	142.37(84.62, 239.53)	142.32(83.84, 241.6)	1992.26	6.98(6.26)	126.4(81.79)	<0.001	<0.001
parathyroid disorder	44	69.62(51.34, 94.41)	69.55(50.83, 95.17)	2799.34	6.03(5.6)	65.55(50.8)	<0.001	<0.001
hyperparathyroidism primary	13	44.71(25.68, 77.83)	44.69(25.81, 77.37)	534.01	5.43(4.66)	43.02(27.05)	<0.001	<0.001
hypoparathyroidism	23	38.04(25.1, 57.64)	38.02(25.19, 57.38)	801.88	5.2(4.61)	36.81(25.99)	<0.001	<0.001
hyperparathyroidism	54	32.54(24.82, 42.66)	32.5(24.7, 42.76)	1602.45	4.98(4.6)	31.62(25.21)	<0.001	<0.001
hyperparathyroidism secondary	34	15.91(11.34, 22.33)	15.9(11.39, 22.19)	468.26	3.97(3.49)	15.7(11.82)	<0.001	<0.001
hypercalcaemia of malignancy	3	14.43(4.62, 45.07)	14.43(4.63, 44.98)	37.02	3.83(2.41)	14.26(5.5)	0.001	0.003
precocious puberty	4	8.41(3.15, 22.5)	8.41(3.16, 22.41)	25.93	3.06(1.79)	8.36(3.67)	0.001	0.004
**Gastrointestinal Disorders**								
pancreatic atrophy	3	9.09(2.92, 28.31)	9.09(2.92, 28.33)	21.42	3.17(1.75)	9.02(3.49)	0.005	0.011
gastrointestinal tract irritation	6	6.84(3.07, 15.27)	6.84(3.06, 15.28)	29.74	2.77(1.69)	6.81(3.48)	<0.001	0.001
abdominal discomfort	755	5.66(5.27, 6.08)	5.59(5.17, 6.05)	2836.13	2.48(2.37)	5.56(5.24)	<0.001	<0.001
oesophagitis ulcerative	3	5.28(1.7, 16.41)	5.28(1.69, 16.46)	10.35	2.39(0.98)	5.26(2.04)	0.02	0.04
gastrointestinal disorder	271	4(3.55, 4.51)	3.99(3.55, 4.49)	604.98	1.99(1.82)	3.98(3.6)	<0.001	<0.001
vomiting	1455	3.92(3.72, 4.13)	3.83(3.61, 4.06)	3056.32	1.93(1.86)	3.82(3.66)	<0.001	<0.001
nausea	2165	3.49(3.34, 3.64)	3.37(3.24, 3.5)	3652.79	1.75(1.69)	3.37(3.25)	<0.001	<0.001
abdominal pain upper	517	3.13(2.87, 3.41)	3.11(2.88, 3.36)	739.44	1.63(1.51)	3.1(2.88)	<0.001	<0.001
dysphagia	234	3.05(2.68, 3.47)	3.04(2.65, 3.49)	319.83	1.6(1.42)	3.03(2.72)	<0.001	<0.001
**General Disorders and Administration Site Conditions**								
calcinosis	20	10.8(6.95, 16.78)	10.8(7.02, 16.62)	176.11	3.42(2.8)	10.7(7.4)	<0.001	<0.001
cardiac death	6	5.92(2.65, 13.21)	5.92(2.65, 13.22)	24.4	2.56(1.49)	5.89(3.01)	0.001	0.002
malaise	1499	4.21(4, 4.43)	4.11(3.88, 4.36)	3542.44	2.04(1.96)	4.1(3.93)	<0.001	<0.001
**Infections and Infestations**								
shunt infection	5	26.69(10.99, 64.79)	26.69(11.05, 64.48)	120.74	4.71(3.53)	26.09(12.42)	<0.001	<0.001
arteriovenous fistula site infection	3	25.47(8.11, 80)	25.47(8.17, 79.38)	68.97	4.64(3.21)	24.93(9.57)	<0.001	0.001
renal cyst infection	3	25.09(7.99, 78.79)	25.09(8.05, 78.2)	67.88	4.62(3.18)	24.56(9.43)	<0.001	<0.001
cardiac valve vegetation	3	7.37(2.37, 22.95)	7.37(2.36, 22.97)	16.42	2.87(1.45)	7.33(2.84)	0.008	0.018
infected fistula	3	7.06(2.27, 21.98)	7.06(2.27, 22)	15.52	2.81(1.39)	7.03(2.72)	0.01	0.021
peritonitis	56	3.28(2.52, 4.26)	3.27(2.53, 4.22)	88.18	1.71(1.33)	3.27(2.62)	<0.001	<0.001
**Cardiac Disorders**								
foetal heart rate deceleration abnormality	6	10.95(4.9, 24.48)	10.95(4.9, 24.46)	53.74	3.44(2.37)	10.86(5.54)	<0.001	<0.001
mitral valve calcification	3	5.68(1.83, 17.66)	5.68(1.82, 17.7)	11.51	2.5(1.08)	5.66(2.19)	0.016	0.034
mitral valve stenosis	3	5.27(1.7, 16.39)	5.27(1.69, 16.43)	10.33	2.39(0.97)	5.25(2.03)	0.02	0.039
arrhythmia	125	3.16(2.65, 3.77)	3.16(2.65, 3.77)	183.78	1.66(1.4)	3.15(2.72)	<0.001	<0.001
**Skin and Subcutaneous Tissue Disorders**								
angiodermatitis	3	45.44(14.33, 144.13)	45.44(14.3, 144.43)	125.3	5.45(4)	43.71(16.64)	<0.001	<0.001
**Respiratory, Thoracic and Mediastinal Disorders**								
increased viscosity of bronchial secretion	3	6.14(1.97, 19.08)	6.14(1.97, 19.14)	12.83	2.61(1.19)	6.11(2.36)	0.014	0.029
**Immune System Disorders**								
multisystem inflammatory syndrome	3	24.02(7.65, 75.38)	24.02(7.71, 74.87)	64.79	4.56(3.12)	23.53(9.04)	<0.001	0.001
**Blood and Lymphatic System Disorders**								
monocytopenia	3	43.67(13.78, 138.4)	43.67(13.74, 138.8)	120.38	5.39(3.95)	42.07(16.02)	<0.001	<0.001

These were categorized into SOCs, revealing that nausea, vomiting, decreased appetite, abdominal discomfort, hypocalcemia, upper abdominal pain, and prolonged QT interval on ECG were among the most frequently reported and exhibited strong signals, all of which are consistent with the drug’s labeling. In addition to the side effects mentioned in the instructions, adverse events such as decreased blood albumin, parathyroid hemorrhage, increased human chorionic gonadotropin, parathyroid cyst, precocious puberty, pancreatic atrophy, cardiac death, cardiac valve vegetation, foetal heart rate deceleration abnormality, mitral valve calcification or stenosis, arrhythmia and monocytopenia were also observed in a certain proportion, which warrants further attention.

## 5. Discussion

This study analyzed adverse event (AE) reports related to cinacalcet from the FAERS database, aiming to provide a new reference evidence for its clinical application through the analysis of real-world data. The collected reports indicate that cinacalcet-associated adverse events were more prevalent in female patients, with a median age of 60 years, and in cases where the drug was administered for <7 days or ≥60 days. Firstly, in terms of gender distribution, since some reports did not include gender information, this study primarily analyzed gender-based distribution characteristics of adverse drug reactions using data with explicitly documented gender (male and female). The analysis revealed that among patients with known gender, the reporting rate of adverse events was significantly higher in females than in males. It should be noted that this distribution pattern is likely related to the higher prevalence of cinacalcet’s indication—primary hyperparathyroidism (PHPT)—in females, particularly postmenopausal women (with an incidence rate of approximately 1–2% in this population in the United States [16,17]). However, the available data only reflect a difference in reporting rates between genders and do not indicate a higher absolute risk of medication use in female patients.. Additionally, differences in lifestyle or genetic factors between genders may result in varied responses to medications. In terms of age distribution, the highest incidence of cinacalcet-related adverse events was observed in the 60–74 age group, followed by the 45–59 age group. Several factors may contribute to this finding: Firstly, this trend is more likely to reflect the natural distribution characteristics of primary hyperparathyroidism in the population. Specifically, the patient population is predominantly middle-aged and elderly, with a particularly high prevalence among women over 45 years of age. Consequently, this demographic exhibits a higher frequency of cinacalcet use [17]. Secondly, middle-aged and elderly patients are relatively weak and often suffer from multiple diseases, which may require them to take multiple medications, and drug interactions in these patients may increase the risk of adverse events [18–20]. Advancing age leads to progressive deterioration of organ function, particularly affecting the metabolic capacity of the liver and kidneys. This physiological decline may result in impaired drug clearance and consequently elevate the risk of adverse reactions [21–24]. Therefore, when treating this specific population, clinicians should thoroughly assess patients’ overall health status before prescribing cinacalcet and closely monitor for potential adverse events during therapy. Analysis of the time period for AE occurrence showed that most adverse events were reported within the first 7 days and at ≥60 days after administration.

Adverse events occurring within 7 days may indicate heightened sensitivity to the drug during the initial phase of treatment [25–27]. This finding underscores the importance of enhanced follow-up observation during the early stages of therapy to promptly detect and manage potential adverse reactions, ensuring the safety and effectiveness of systematic treatment. On the other hand, adverse events emerging after 60 days of administration may be associated with the patient’s underlying pathological conditions, which can influence the manifestation and timing of drug-related adverse effects, and this is particularly relevant for chronic disease patients (e.g., CKD), who may experience disease-related adverse events only after prolonged medication use [28,29]. Consequently, enhanced follow-up observation should also be implemented for this patient group during long-term therapy. Geographically, the majority of AE reports originated from the United States, accounting for 89.52% of the total reports, and this suggests a potential correlation between a country’s development level and its emphasis on drug safety, while also serving as a warning for other countries to strengthen the monitoring and reporting of adverse drug events [30].

During the signal mining of cinacalcet adverse events, we found that many adverse events were consistent with the drug label, such as “hypocalcemia, ECG showing QT prolongation, nausea, vomiting, and abdominal discomfort.” These PTs had high report counts and strong signals, all aligning with the descriptions in the current prescribing information.

Hypocalcemia is one of the most common adverse events associated with cinacalcet in the treatment of hyperparathyroidism. Multiple studies have indicated a high incidence of hypocalcemia in patients receiving cinacalcet [31–34]. For instance, the systematic review by Ballinger et al [32]demonstrated an increased risk of hypocalcemia in dialysis adults receiving cinacalcet. Similarly, the review by Warady et al [5]reported that the most frequent adverse events were primarily hypocalcemia(22.8%), vomiting(16.5%), nausea(15.2%), systemic hypertension(11.4%), and muscle cramps(10.1%). Schmidt GS et al [33]also described a case of severe symptomatic hypocalcemia complicated by arrhythmia in a patient with primary hyperparathyroidism during cinacalcet therapy. In addition to the aforementioned findings, the results of this study also indicate that “tetany” constitutes a strong signal event. It is speculated that this symptom is likely induced by hypocalcemia, thereby further confirming that hypocalcemia is one of the common adverse events associated with cinacalcet. Regarding the underlying mechanism, we propose that the occurrence of hypocalcemia may be closely related to the pharmacological mechanism of cinacalcet.

Specifically, cinacalcet inhibits PTH secretion by suppressing cell proliferation, reducing cell number and parathyroid size, and decreasing PTH gene transcription. Additionally, it activates local synthesis of 1,25(OH)_2_D_3_ (an inhibitor of PTH synthesis) in the oxyphil cells of the parathyroid glands. Therefore, prolonged treatment with cinacalcet can inhibit parathyroid cell proliferation and lower serum PTH levels, which may significantly increase the risk of hypocalcemia in susceptible patients [34]. Furthermore, Temiz et al [35]demonstrated a correlation between the therapeutic dosage of cinacalcet and QT interval prolongation. Novick et al [36] also described a case of hypocalcemia, torsade de pointes, and cardiac arrest after cinacalcet treatment in a middle-aged man with secondary hyperparathyroidism. To prevent the occurrence of such serious adverse events, patients should be closely monitored for renal function and electrolyte levels (especially serum calcium) during treatment. If abnormal findings suggest severe hypocalcemia (such as depressed mood, muscle cramps, blood pressure drops, prolongation of the QT interval on electrocardiography, or arrhythmias such as premature ventricular contractions), serum calcium concentration should be measured immediately. Depending on the results, interventions such as calcium/vitamin D supplementation, dose adjustment of cinacalcet, or temporary treatment discontinuation should be implemented to prevent worsening of adverse effects.

Several studies [37–40] have found that one of the most common adverse events of cinacalcet in the treatment of hyperparathyroidism is gastrointestinal (GI) symptoms, such as nausea, vomiting, loss of appetite, and abdominal discomfort. These symptoms are also the main reasons for drug discontinuation. For example, a phase III randomized double-blind trial [37] reported a 32.8% incidence of gastrointestinal adverse events in the cinacalcet group, primarily manifested as nausea (14.2%), vomiting (11.7%), diarrhea (9.5%), and abdominal discomfort (11%). The study found that the majority of patients discontinued or interrupted medication due to severe gastrointestinal reactions and intolerance [38]. A systematic review and meta-analysis conducted by Suetonia C Palmer et al [38] also found that common AEs associated with cinacalcet included gastrointestinal symptoms such as nausea, vomiting, and diarrhea, and these symptoms may adversely affect patients’ nutritional status and quality of life to some extent. Additionally, Otsuka K et al [39]found that among patients with secondary hyperparathyroidism undergoing hemodialysis, those with gallstones were more likely to experience gastrointestinal adverse events from cinacalcet, primarily nausea and vomiting, which was presumed to be related to biliary dyskinesia. Moreover, Schaefer et al [40]found that cinacalcet demonstrated comparable efficacy whether administered with the first full meal after dialysis or during dialysis, with both dosing timings showing high bioavailability and good tolerability. Notably, evening intake appeared to be associated with a lower incidence of gastrointestinal adverse events. To reduce gastrointestinal adverse events in patients with secondary hyperparathyroidism (SHPT), it is recommended to perform abdominal ultrasound screening for gallstones before initiating cinacalcet treatment and administer the drug in the evening with or after meals or in combination with a gastrointestinal protective agent. If mild nausea, vomiting, or abdominal discomfort occurs, these symptoms can be managed under the guidance of a physician. In cases of serious adverse events, such as peptic ulcer or gastrointestinal bleeding, the drug should be discontinued immediately.

It is noteworthy that some new signals of adverse events were found in this study, such as pancreatic atrophy, dysphagia, hypoproteinemia in gastrointestinal diseases, and precocious puberty, parathyroid cyst, and parathyroid hemorrhage in endocrine diseases.

In this study, precocious puberty (ROR = 8.41, PRR = 8.41, IC025 = 3.06, EBGM05 = 8.36) was identified as a strong positive signal. There have been several cases of patients showing signs of precocity after taking cinacalcet. For instance, it was first reported that a 5-year-old child with severe chronic kidney disease caused by a de novo TCF2/HNF1β gene mutation (renal cyst and diabetes syndrome) developed signs of precocious puberty (bilateral testicular enlargement and increased penis length) after taking cinacalcet for 2 weeks [41]. The biological and clinical abnormalities gradually disappeared after discontinuation of the drug. It was speculated that the drug may disrupt a pre-existing abnormal endocrine disorder, such as premature activation of the LH receptor, and that cinacalcet may activate both the therapeutic target CaR and the LH receptor simultaneously, thereby inducing hypertestosteronemia and clinical symptoms [41]. However, the mechanistic link between precocious puberty and cinacalcet treatment remains unclear. This suggests that physicians should pay more attention to this adverse event during the diagnosis and treatment of children. For patients experiencing genital changes after receiving cinacalcet or other calcimimetics, the drug should be used judiciously with close monitoring of plasma testosterone levels. In addition, Bernardor J et al [42]found in a study that three children developed precocious puberty, two of whom underwent genetic testing suggestive of a pathogenic HNF1β gene mutation.

Additionally, “increased human chorionic gonadotropin” was identified as a strong new signal in this study. Based on these findings, it is recommended that changes in sex hormone levels in children treated with cinacalcet be closely monitored in clinical practice, especially in patients with HNF1β gene mutations [42]. In conclusion, precocious puberty may represent a new adverse event associated with cinacalcet, but its specific mechanism has not been systematically studied. Future research should elucidate the pathogenesis and preventive strategies for this complication.

In addition to the precocious puberty mentioned above, it is noteworthy that this study has also identified parathyroid hemorrhage as a distinctive signal. Parathyroid hemorrhage is a relatively rare clinical condition characterized by abnormal bleeding within the parathyroid gland, often caused by the rupture of a parathyroid adenoma or trauma. In 2012, Nagasawa M et al [43] reported a case of parathyroid hemorrhage in a patient with secondary hyperparathyroidism after taking cinacalcet. This patient was admitted with respiratory distress, parathyroid hormone levels decreased after taking cinacalcet but developed massive bleeding. During surgery, ruptured parathyroid glands with nodular hyperplasia were identified.

However, the exact cause of parathyroid hemorrhage after cinacalcet administration is unclear. Based on previous reports and findings, it has been hypothesized that two types of hyperplastic parathyroid cells exist: cinacalcet-responsive and non-responsive variants. The pharmacological reduction of responsive cells may diminish intracapsular pressure, consequently diverting excessive blood flow toward non-responsive cell clusters. When this compensatory perfusion surpasses the vascular compliance limit, parenchymal rupture and subsequent hemorrhage may ensue. Thus, parathyroid hemorrhage may be a result of glandular degeneration induced by cinacalcet [44]. When discussing the pathological manifestations of parathyroid hemorrhage, particular attention should be given to a more distinct clinical emergency—parathyroid apoplexy, which refers to hemorrhage or necrosis within a parathyroid adenoma, and it may occur spontaneously or result from trauma/surgical intervention. Di Dalmazi G et al [44]reported a case of parathyroid apoplexy following cinacalcet administration in a patient with primary hyperparathyroidism. Parathyroid apoplexy exhibits heterogeneous manifestations, ranging from asymptomatic cases to life-threatening emergencies. Clinicians should be alert to symptoms such as neck swelling, pain, dysphagia, cough and dyspnea, which may indicate parathyroid hemorrhage or apoplexy. Early recognition and intervention are critical to preventing severe outcomes.

This study has identified several potential adverse event signals not currently mentioned in either the current drug labels or clinical studies, including hypoalbuminemia, peritonitis, pancreatic atrophy, and monocytopenia, as well as significant high-risk cardiovascular signals such as cardiac death, cardiac valve vegetation, foetal heart rate deceleration abnormality, mitral valve calcification or stenosis, and arrhythmia, etc. None of these signals have been reported in the current labeling or existing literature, warranting heightened clinical attention. In particular, “hypoproteinemia” appeared frequently in the data. While no direct evidence establishes cinacalcet as the cause of decreased protein levels, this study hypothesizes two potential mechanisms based on the findings of this investigation: first, pre-existing liver dysfunction in treated patients (including hepatitis, cirrhosis, alcoholic liver disease, or hepatocellular carcinoma); second, potential association with chronic gastrointestinal adverse events like nausea, vomiting, abdominal discomfort, and loss of appetite that may affect nutritional absorption.

In conclusion, the current discussion on hypoproteinemia as an adverse event of cinacalcet remains speculative. Further experimental and clinical studies are required to clarify the causal relationship. In the interim, healthcare professionals must maintain meticulous monitoring and rigorous reporting practices in clinical practice, implementing timely interventions when clinically indicated.

## 6. Limitations

This study has several limitations. Firstly, the data involved in this study were primarily obtained from the FAERS database, which is large and covers a wide range of populations, but there is a risk of incomplete data capture, underreporting, repeated reporting, and inaccurate reporting, so these factors may introduce bias into the study results. Particularly, the lack of accurate denominator data for drug-exposed populations (i.e., the total number of patients taking the target drug) precludes calculation of the actual incidence rates of adverse events. For example, although the 60–74 age group reported a higher number of adverse events, this may merely reflect a higher degree of drug exposure in this population rather than a true risk difference. In addition, the high rate of missing data for drug start dates or (and) event occurrence dates in the database precluded the use of formal time-to-event models, as their application would have led to biased results due to data incompleteness. Secondly, due to the lack of data on the number of patients who did not experience adverse events while taking cinacalcet, the incidence of adverse events related to cinacalcet could not be calculated. Thirdly, the database lacks long-term safety evaluations of cinacalcet. The signal detection methods employed (e.g., ROR, PRR) only measure the strength of association between cinacalcet and adverse events, indicating statistical correlations rather than establishing biological causality. Consequently, more rigorous clinical observation and research are urgently needed to determine whether there is a biological causal relationship between cinacalcet and the adverse events identified in this study.

## 7. Conclusions

In conclusion, this study provided a scientific basis for the safety evaluation of cinacalcet by mining adverse event report data from the FAERS database and analyzing the data using multiple algorithms at different levels. This study not only identified known adverse events, such as electrolyte disturbances (e.g., hypocalcemia), gastrointestinal diseases, and cardiovascular disorders, but also uncovered new signals, including precocious puberty, parathyroid hemorrhage, hypoproteinemia, monocytopenia, cardiac death and arrhythmia, which were not mentioned in the manufacturer’s instructions. Although further researches are required to establish definitive causal relationships, these findings provide corresponding value for future pharmacovigilance research and regulatory safety monitoring, while also offering targeted guidance for optimizing clinical medication safety practices.

## Supporting information

S1 Table The generic name cinacalcet (brand name Sensipar) was used as the search term for the target drug. This table details the specific search strategy employed to identify the target drug in the FAERS database, including both the generic and brand names used for comprehensive retrieval of relevant case reports.
(DOCX)

S2 Table Distribution of AEs of cinacalcet from 2004 to the first quarter of 2025 (Q1). This table presents the temporal distribution of cinacalcet-related adverse events in the FAERS database from 2004 to the first quarter of 2025, showing quarterly reporting frequencies and trend patterns.
(DOCX)

S3 Table The signal strength of AEs of cinacalcet at the SOC level in FAERS database. This table presents the signal strength analysis of cinacalcet-associated adverse events at the System Organ Class (SOC) level, demonstrating disproportionality analysis results derived from the FAERS database, including statistical measures and significance indicators across SOC categories.
(DOCX)

## Abbreviations

AEs: adverse events
FAERS: the U.S. Food and Drug Administration Adverse Event Reporting System
PTs: preferred terms
SOCs: system organ classes
BCPNN: Bayesian confidence propagation neural network
EBGM: empirical Bayesian geometric mean
ROR: reporting odds ratio
PRR: proportional reporting ratio
95% CI: 95% confidence interval
PS: primary suspect
SS: secondary suspect
FDA: food and drug administration
EMA: European Medicines Agency
MedDRA: the Medical Dictionary for Regulatory Activities
PTH: parathyroid hormone
HPT: hyperparathyroidism
SHPT: secondary hyperparathyroidism
PHPT: primary hyperparathyroidism
THPT: tertiary hyperparathyroidism
CaSR: calcium-sensing receptor
TTO: time to onset
