# Supplementary material for: Safety evaluation of cinacalcet: Signal mining and analysis of adverse events based on the FAERS database
Source: PLoS One. 2025 Oct 27;20(10):e0331510. doi: 10.1371/journal.pone.0331510 (PMC12558480; doi:10.1371/journal.pone.0331510)
Supplement: S1 Table — This table details the specific search strategy employed to identify the target drug in the FAERS database, including both the generic and brand names used for comprehensive retrieval of relevant case reports. (DOCX) [file pone.0331510.s001.docx]

S1 Table.The generic name cinacalcet (brand name Sensipar) was used as the search term for the target drug.

| drugname(54582) | prod_ai | number of AEs |
| --- | --- | --- |
| sensipar | cinacalcet hydrochloride | 45986 |
| mimpara | cinacalcet hydrochloride | 2460 |
| regpara | cinacalcet hydrochloride | 1727 |
| cinacalcet hcl | cinacalcet hydrochloride | 1309 |
| cinacalcet hydrochloride | cinacalcet hydrochloride | 1217 |
| cinacalcet | cinacalcet | 747 |
| cinacalcet. | cinacalcet | 482 |
| cinacalcet hydrochloride. | cinacalcet hydrochloride | 190 |
| sensipar (cinacalcet hydrochloride) | sensipar (cinacalcet hydrochloride) | 73 |
| cinacalcet hcl - khk | cinacalcet hydrochloride | 41 |
| cinacalcet hydrochloride (cinacalcet hydrochloride) | cinacalcet hydrochloride (cinacalcet hydrochloride) | 19 |
| 70436-007-04 cinacalcet hydrochloride tablet 30mg | cinacalcet hydrochloride | 18 |
| cinacalcet (cinacalcet) | cinacalcet | 13 |
| cinacalcet/cinacalcet hydrochloride | cinacalcet\cinacalcet hydrochloride | 11 |
| cinacalcet hcl 30mg cipla usa | cinacalcet hydrochloride | 11 |
| sensipar (cinacalcet) | sensipar (cinacalcet) | 10 |
| mimpara (cinacalcet) | cinacalcet | 7 |
| cinacalcet film coated tablets | cinacalcet hydrochloride | 7 |
| mimpara [cinacalcet hydrochloride] | cinacalcet hydrochloride | 6 |
| cinacalcet - blinded | cinacalcet | 6 |
| cinacalet | cinacalcet | 6 |
| regpara (cinacalcet hydrochloride) tablet | cinacalcet hcl | 5 |
| cinacalcet film coated tablet | cinacalcet hydrochloride | 5 |
| cinacalcet hydrochloride tablet | cinacalcet hydrochloride | 5 |
| mimpara (cinacalcet hydrochloride) | mimpara (cinacalcet hydrochloride) | 5 |
| mimpara [cinacalcet] | cinacalcet | 5 |
| cinacalcet (mimpara) | cinacalcet | 5 |
| cinacalcet (sensipar) | cinacalcet (sensipar) | 5 |
| apo-cinacalcet | cinacalcet | 5 |
| cinacalcet hydrochloride (cinacalcet hydrochloride) tablet | cinacalcet hcl | 4 |
| cinacalcet hydrochloride (regpara) (cinacalcet hydrochloride) tablet | cinacalcet hcl | 4 |
| cinacalet hcl 30mg cipla usa | cinacalcet hydrochloride | 4 |
| regpara (cinacalcet hydrochloride) | regpara (cinacalcet hydrochloride) | 4 |
| mimpara 30 mg | cinacalcet hydrochloride | 4 |
| mimpara 30 mg, comprime pellicule | cinacalcet hydrochloride | 4 |
| sensipar (cinacalcet hydrochloride) (unknown) | cinacalcet | 3 |
| cinacalcet [cinacalcet hydrochloride] | cinacalcet hydrochloride | 3 |
| sensipar(cinacalcet hydrochloride) | sensipar(cinacalcet hydrochloride) | 3 |
| cinacalcet zentiva | cinacalcet | 3 |
| cinacalcet hcl 30mg | cinacalcet hydrochloride | 3 |
| cinacalcet hcl ? khk | cinacalcet hydrochloride | 3 |
| regpara tablets | cinacalcet hydrochloride | 3 |
| sensipar tablet | cinacalcet | 3 |
| cinacalcet 30mg | cinacalcet | 3 |
| cincalcet hcl 30mg cipla usa | cinacalcet hydrochloride | 3 |
| cinacalcet arrow | cinacalcet | 3 |
| senispar (cinacalcet hydrochloride) | cinacalcet hcl | 2 |
| sensipar (cinacalet hydrochloride) | hydrochloride; cinacalcet | 2 |
| senispar (interpreted as sensipar) | cinacalcet | 2 |
| sensipar (cinacalcet hydrochloride) unknown | cinacalcet | 2 |
| mimpara 60 mg, comprime pellicule | cinacalcet hydrochloride | 2 |
| mimpara 30 mg, comprim? pellicul? | cinacalcet hydrochloride | 2 |
| cinacalcet hcl 30 mg tab | cinacalcet | 2 |
| mimpara (cinacalcet hcl) | cinacalcet hcl | 2 |
| cinacalcet(cinacalcet) | cinacalcet | 2 |
| mimpara / cinacalcet | cinacalcet | 2 |
| cinacalcet hcl 30 mg | cinacalcet hydrochloride | 2 |
| minipara (cinacalcet) | cinacalcet | 2 |
| blinded cinacalcet hcl | cinacalcet hydrochloride | 2 |
| cinacalcet 60mg | cinacalcet | 2 |
| cinacalcet, 30mg | cinacalcet | 2 |
| cinacalcet aurovitas 30 mg film coated tablet efg | cinacalcet | 2 |
| cinacalcet hcl 60mg tab (cinacalcet) | cinacalcet; cinacalcet hydrochloride | 2 |
| cinacalet hcl 60mg cipla usa | cinacalcet hydrochloride | 2 |
| cinacalcet dr. reddys | cinacalcet | 2 |
| cinacalcet hcl 60 mg | cinacalcet hydrochloride | 2 |
| cinacalcet (sensipar) 30 mg tablet | cinacalcet | 2 |
| cinacalcet tab | cinacalcet | 2 |
| cinacalcet teva | cinacalcet | 2 |
| mylan cinacalcet | cinacalcet | 2 |
| sensipar (cinacalcet hydrochloride, cinacalcet) | sensipar (cinacalcet hydrochloride, cinacalcet) | 1 |
| 70436-008-04 cinacalcet hydrochloride tablet 60mg | cinacalcet hydrochloride | 1 |
| cinacalcet hydrochloride (cinacalcet hydrochlorie) | cinacalcet; cinacalcet hcl | 1 |
| cinacalcet hydrochloride(cinacalcet hydrochloride) | cinacalcet hcl | 1 |
| cinacalet hydrochloride (cinacalcet hydrochloride) | hydrochloride | 1 |
| cinacalcet hydrochloride 9cinacalcet hydrochlooride) | cinacalcet hcl | 1 |
| cinacalcet hydrochloride oral tablet (generic sensipar0 | cinacalcet hydrochloride | 1 |
| cinacalcet hydrochloride 60 mg tab slat ndc:70436-0008-04 | cinacalcet hydrochloride | 1 |
| sensipar (cinacalcet hydrochloride) (90 milligram, tablets) | cinacalcet | 1 |
| mimpara (cinacalcet hydrochloride) ?(cinacalcet hydrochloride) | mimpara (cinacalcet hydrochloride) ?(cinacalcet hydrochloride) | 1 |
| dk-ema-dd-20171205-kumarnvevhp-125402 (cinacalcet hydrochloride) | cinacalcet hydrochloride | 1 |
| cinacalcet hydrochloride (cinacalcet hydrochloride) per oral nos | cinacalcet hcl | 1 |
| cinacalcet hydrochloride (regpara)(cinacalcet hydrochloride) tablet | cinacalcet hcl | 1 |
| sensipar (cinacalcet hydrochloride) (30 mill igram) (cinacalcet hydroc | cinacalcet | 1 |
| sensipar (cinacalcet hydrochloride) (30 milligram) (cinacalcet hydroch | cinacalcet | 1 |
| sensipar (cinacalcet hydorchloride) | cinacalcet | 1 |
| cinacalcet (cinacalcet) (cinacalcet) | cinacalcet | 1 |
| sensipar 30 (cinacalcet hydrochloride) | sensipar 30 (cinacalcet hydrochloride) | 1 |
| sensipar (cinacalcet hydrochloride) tablet | cinacalcet | 1 |
| cincalcet hcl30mg slate run pharmaceuticals | cinacalcet hydrochloride | 1 |
| sensipar(cinacalcet hydrochloride) (unknown) | sensipar(cinacalcet hydrochloride) (unknown) | 1 |
| cinacalcet (calcium) (30 milligram) (calcium) | cinacalcet (calcium) (30 milligram) (calcium) | 1 |
| cinacalcet hydrochloride unk | cinacalcet hcl | 1 |
| cinacalcet hcl 60mg cipla usa | cinacalcet hydrochloride | 1 |
| cinacalcet hcl 60mg slate run | cinacalcet hydrochloride | 1 |
| cinacalcet hcl 90mg cipla usa | cinacalcet hydrochloride | 1 |
| cinacalet hcl 90mg cipla usa | cinacalcet hydrochloride | 1 |
| cinacalcet (mimpara) (cinacalcet) | cinacalcet | 1 |
| regpara (cinacalcet hydrochlroide) | regpara (cinacalcet hydrochlroide) | 1 |
| sensipar (cincalcet hydrochloride) | hydrochloride; cinacalcet | 1 |
| sensipar cinacalcet hydrochloride) | cinacalcet hcl; cinacalcet | 1 |
| cinacalcet hydrochloride (regpara) | cinacalcet hcl | 1 |
| cinacalcet hcl 30mg tab | cinacalcet hydrochloride | 1 |
| cinacalcet hci 30mg tab | cinacalcet hydrochloride | 1 |
| cinacalcet hcl tab 30mg | cinacalcet hydrochloride | 1 |
| calcimimetic cinacalcet | calcimimetic cinacalcet | 1 |
| cinacalect (cinacalcet) | cinacalcet | 1 |
| sensipar -paricalcitol.- | ; cinacalcet | 1 |
| cinacalcet hydrochloride) | cinacalcet hcl | 1 |
| cinacalcet unknown | cinacalcet | 1 |
| cinacalcet(mimpera) | cinacalcet(mimpera) | 1 |
| mimpara(cinacalcet) | cinacalcet | 1 |
| sensipar/cinacaclet | cinacalcet | 1 |
| sensipar 30mg tablet | cinacalcet | 1 |
| cinacalcet hcl++ 30mg | cinacalcet hydrochloride | 1 |
| cinacalcet hcl, 30 mg | cinacalcet hydrochloride | 1 |
| cinacalcet 30mg daily | cinacalcet 30mg daily | 1 |
| cinacalcet {sensipar} | cinacalcet | 1 |
| cinacalcet -sensipar- | cinacalcet | 1 |
| cinacalcet 30mg amgen | cinacalcet | 1 |
| cinacalcet/mimpara | cinacalcet | 1 |
| cinacalcet 60mg po qhs | cinacalcet 60mg po qhs | 1 |
| sensipar 30 mg | cinacalcet | 1 |
| cinacalcet hci | cinacalcet | 1 |
| cinacelcet 30mg | cinacalcet hydrochloride | 1 |
| cinacalcet tablet | cinacalcet | 1 |
| cinacalcet, 30 mg | cinacalcet | 1 |
| sensipar 30mg tab | cinacalcet | 1 |
| cinnacalcet | cinnacalcet | 1 |
| cinacacet | cinacacet | 1 |
| cinacalcet hc | cinacalcet hc | 1 |
| viscap | cinacalcet hydrochloride | 1 |
| auro?cinacalcet tablets | cinacalcet | 1 |
| cinacalcet hcl, 30 mg/kg | cinacalcet hydrochloride | 1 |
| cinacalcet 90mg | cinacalcet | 1 |
| cinacalcet hcl 30mg tabs | cinacalcet hydrochloride | 1 |
| cinacalet hcl 30mg tab | cinacalcet hydrochloride | 1 |
| cinacalcet, | cinacalcet | 1 |
| cinacalcet mylan 30 mg film-coated tablets | cinacalcet hydrochloride | 1 |
| cinacalcet hcl 60mg | cinacalcet hydrochloride | 1 |
| cincalet hcl 30mg | cinacalcet hydrochloride | 1 |
| cinacalcet tablets | cinacalcet | 1 |
| cinacalcet (cinacalcet hcl 30mg tab) | cinacalcet hydrochloride | 1 |
| cincalet 30mg | cinacalcet hydrochloride | 1 |
| cinacalcet 30mg tablets | cinacalcet | 1 |
| cinacalcet 30 mg tablet | cinacalcet | 1 |
| cinacalcet hcl 30mg tablets | cinacalcet hydrochloride | 1 |
| cinacalcet hci 30 mg | cinacalcet hydrochloride | 1 |
| cinacalcet dr. reddy?s, 30 mg compresse rivestite con film | cinacalcet | 1 |
| cinacalcet hcl30mg cipla usa | cinacalcet hydrochloride | 1 |
| cinacalcet dr. reddy?s, 90 mg compresse rivestite con film | cinacalcet hydrochloride | 1 |
| cinacalcelt | cinacalcet | 1 |
| cinacalcet arxwp | cinacalcet | 1 |
| cinacalcet hcl 60mg tablets | cinacalcet hydrochloride | 1 |
| cinacalcet mylan | cinacalcet hydrochloride | 1 |
| apo cinacalcet | cinacalcet | 1 |
| cinacalcet stada | cinacalcet | 1 |
| cinacalcet\cinacalcet hydrochloride | cinacalcet\cinacalcet hydrochloride | 1 |
| cinacalcet orion | cinacalcet | 1 |
| cinacalcet 30 mg | cinacalcet | 1 |
| cinacalcet accord | cinacalcet | 1 |
