# Supplementary material for: Safety evaluation of cinacalcet: Signal mining and analysis of adverse events based on the FAERS database
Source: PLoS One. 2025 Oct 27;20(10):e0331510. doi: 10.1371/journal.pone.0331510 (PMC12558480; doi:10.1371/journal.pone.0331510)
Supplement: S2 Table — This table presents the temporal distribution of cinacalcet-related adverse events in the FAERS database from 2004 to the first quarter of 2025, showing quarterly reporting frequencies and trend patterns. (DOCX) [file pone.0331510.s002.docx]

| Year | Reported cases |
| --- | --- |
| 2004 | 97 |
| 2005 | 158 |
| 2006 | 220 |
| 2007 | 145 |
| 2008 | 219 |
| 2009 | 179 |
| 2010 | 202 |
| 2011 | 284 |
| 2012 | 327 |
| 2013 | 696 |
| 2014 | 894 |
| 2015 | 3305 |
| 2016 | 5543 |
| 2017 | 7834 |
| 2018 | 7804 |
| 2019 | 490 |
| 2020 | 419 |
| 2021 | 450 |
| 2022 | 369 |
| 2023 | 387 |
| 2024 | 428 |
| 2025（Q1） | 90 |

S2 Table. Distribution of AEs of cinacalcet from 2004 to the first quarter of 2025 (Q1)
