# Supplementary material for: Safety evaluation of cinacalcet: Signal mining and analysis of adverse events based on the FAERS database
Source: PLoS One. 2025 Oct 27;20(10):e0331510. doi: 10.1371/journal.pone.0331510 (PMC12558480; doi:10.1371/journal.pone.0331510)
Supplement: S3 Table — This table presents the signal strength analysis of cinacalcet-associated adverse events at the System Organ Class (SOC) level, demonstrating disproportionality analysis results derived from the FAERS database, including statistical measures and significance indicators across SOC categories. (DOCX) [file pone.0331510.s003.docx]

S3 Table. The signal strength of AEs of cinacalcet at the SOC level in FAERS database

| **SOCs** | **Case Reports** | **ROR(95% CI)** | **PRR(95% CI)** | **χ2** | **IC(IC025)** | **EBGM(EBGM05)** |
| --- | --- | --- | --- | --- | --- | --- |
| Investigations | 7793 | 2.84(2.77, 2.91) | 2.54(2.49, 2.59) | 7735.24 | 1.34(1.31) | 2.53(2.48) |
| Metabolism and nutrition disorders | 2855 | 2.78(2.68, 2.89) | 2.68(2.58, 2.79) | 3058.95 | 1.42(1.36) | 2.67(2.59) |
| Endocrine disorders | 315 | 2.49(2.23, 2.78) | 2.48(2.2, 2.79) | 277.76 | 1.31(1.15) | 2.47(2.26) |
| Gastrointestinal disorders | 7930 | 2.04(1.99, 2.09) | 1.86(1.82, 1.9) | 3479.91 | 0.9(0.86) | 1.86(1.83) |
| Injury, poisoning and procedural complications | 6178 | 1.35(1.32, 1.39) | 1.31(1.28, 1.34) | 489.37 | 0.38(0.35) | 1.31(1.28) |
| General disorders and administration site conditions | 9086 | 1.06(1.03, 1.08) | 1.05(1.03, 1.07) | 23.98 | 0.07(0.03) | 1.05(1.03) |
| Vascular disorders | 1036 | 0.97(0.91, 1.03) | 0.97(0.91, 1.03) | 1.27 | -0.05(-0.14) | 0.97(0.92) |
| Cardiac disorders | 1269 | 0.96(0.91, 1.02) | 0.97(0.91, 1.03) | 1.61 | -0.05(-0.13) | 0.97(0.92) |
| Musculoskeletal and connective tissue disorders | 2070 | 0.78(0.74, 0.81) | 0.79(0.76, 0.82) | 124.92 | -0.34(-0.41) | 0.79(0.76) |
| Pregnancy, puerperium and perinatal conditions | 142 | 0.67(0.57, 0.79) | 0.67(0.57, 0.78) | 23.53 | -0.58(-0.82) | 0.67(0.58) |
| Nervous system disorders | 2727 | 0.63(0.6, 0.65) | 0.65(0.63, 0.68) | 569.88 | -0.62(-0.68) | 0.65(0.63) |
| Infections and infestations | 1581 | 0.59(0.56, 0.62) | 0.6(0.58, 0.62) | 449.42 | -0.74(-0.81) | 0.6(0.57) |
| Renal and urinary disorders | 517 | 0.56(0.52, 0.61) | 0.57(0.53, 0.62) | 173.91 | -0.82(-0.94) | 0.57(0.53) |
| Ear and labyrinth disorders | 95 | 0.44(0.36, 0.54) | 0.44(0.36, 0.54) | 66.79 | -1.17(-1.46) | 0.44(0.37) |
| Immune system disorders | 215 | 0.38(0.34, 0.44) | 0.39(0.34, 0.45) | 211.29 | -1.37(-1.56) | 0.39(0.35) |
| Skin and subcutaneous tissue disorders | 1061 | 0.38(0.36, 0.4) | 0.39(0.37, 0.41) | 1048.25 | -1.34(-1.43) | 0.39(0.37) |
| Neoplasms benign, malignant and unspecified (incl cysts and polyps) | 504 | 0.38(0.35, 0.41) | 0.39(0.36, 0.42) | 508.35 | -1.38(-1.5) | 0.39(0.36) |
| Respiratory, thoracic and mediastinal disorders | 922 | 0.38(0.35, 0.4) | 0.39(0.37, 0.41) | 938.34 | -1.37(-1.46) | 0.39(0.37) |
| Hepatobiliary disorders | 168 | 0.36(0.31, 0.42) | 0.37(0.32, 0.43) | 184.88 | -1.44(-1.66) | 0.37(0.32) |
| Psychiatric disorders | 760 | 0.26(0.24, 0.28) | 0.27(0.25, 0.29) | 1600.39 | -1.89(-1.99) | 0.27(0.25) |
| Eye disorders | 244 | 0.24(0.21, 0.27) | 0.24(0.21, 0.27) | 585.61 | -2.04(-2.22) | 0.24(0.22) |
| Blood and lymphatic system disorders | 197 | 0.23(0.2, 0.26) | 0.23(0.2, 0.26) | 511.77 | -2.11(-2.31) | 0.23(0.21) |
| Congenital, familial and genetic disorders | 27 | 0.18(0.12, 0.26) | 0.18(0.12, 0.26) | 104.56 | -2.51(-3.04) | 0.18(0.13) |
| Reproductive system and breast disorders | 63 | 0.15(0.12, 0.2) | 0.15(0.12, 0.19) | 294.03 | -2.69(-3.05) | 0.15(0.13) |
